# A robotic system for solo surgery in flexible ureteroscopy: development and evaluation with clinical users

**DOI:** 10.1007/s11548-023-02883-5

**Published:** 2023-04-09

**Authors:** Christopher Schlenk, Katharina Hagmann, Florian Steidle, Laura Oliva Maza, Alexander Kolb, Anja Hellings-Kuß, Dominik Stefan Schöb, Julian Klodmann, Arkadiusz Miernik, Alin Albu-Schäffer

**Affiliations:** 1 German Aerospace Center (DLR), Institute of Robotics and Mechatronics, Wessling, Germany; 2 Department of Urology, Faculty of Medicine, University of Freiburg – Medical Centre, Freiburg im Breisgau, Germany

**Keywords:** Flexible ureteroscopy (fURS), Kidney stones, Surgical robot, Physical human-robot interaction, DLR MIRO, User study

## Abstract

**Purpose:**

The robotic system *CoFlex* for kidney stone removal via flexible ureteroscopy (fURS) by a single surgeon (solo surgery, abbreviated SSU) is introduced. It combines a versatile robotic arm and a commercially available ureteroscope to enable gravity compensation and safety functions like virtual walls. The haptic feedback from the operation site is comparable to manual fURS, as the surgeon actuates all ureteroscope DoF manually.

**Methods:**

The system hardware and software as well as the design of an exploratory user study on the simulator model with non-medical participants and urology surgeons are described. For each user study task both objective measurements (e.g., completion time) and subjective user ratings of workload (using the NASA-TLX) and usability (using the System Usability Scale SUS) were obtained.

**Results:**

*CoFlex* enabled SSU in fURS. The implemented setup procedure resulted in an average added setup time of 341.7 ± 71.6 s, a NASA-TLX value of 25.2 ± 13.3 and a SUS value of 82.9 ± 14.4. The ratio of inspected kidney calyces remained similar for robotic (93.68 %) and manual endoscope guidance (94.74 %), but the NASA-TLX values were higher (58.1 ± 16.0 vs. 48.9 ± 20.1) and the SUS values lower (51.5 ± 19.9 vs. 63.6 ± 15.3) in the robotic scenario. SSU in the fURS procedure increased the overall operation time from 1173.5 ± 355.7 s to 2131.0 ± 338.0 s, but reduced the number of required surgeons from two to one.

**Conclusions:**

The evaluation of *CoFlex* in a user study covering a complete fURS intervention confirmed the technical feasibility of the concept and its potential to reduce surgeon working time. Future development steps will enhance the system ergonomics, minimize the users’ physical load while interacting with the robot and exploit the logged data from the user study to optimize the current fURS workflow.

**Supplementary Information:**

The online version contains supplementary material available at 10.1007/s11548-023-02883-5.

## Introduction

Kidney stones are a widespread disease worldwide: 7–13% of the people in North America, 5–9% of the Europeans and 1–5% of the Asians experience kidney stones during their lifetime [1].

**Fig. 1:**
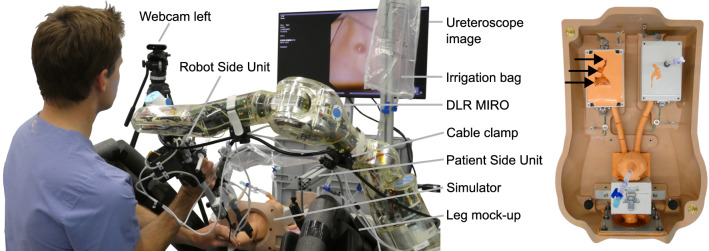
Setup in the robotic scenario of Task 3: the surgeon manipulates the robot side unit with the flexible ureteroscope to remove kidney stones from the simulator. The leg mock-ups limit the motion range for surgeon and robotic system like in a real operating room. The simulator (right) contained one kidney stone per calyx group (arrows)

Treatment options depend on the size, location and composition of the stones [2]. One common method is flexible ureteroscopy (fURS) as it has few contraindications and is applicable in the whole urinary tract [2, 3]. In fURS, a flexible ureteroscope (FU; specialized endoscope for the urinary tract with one bending degree of freedom (DoF)) is advanced through the urinary tract to the stone location. Then, the stone is either captured directly using forceps or a basket, or it is first fragmented by a laser. All end effectors (EEs) are inserted through the endoscope’s working channel. An ureteral access sheath (UAS) can be inserted to simplify the access to the upper urinary tract [2] (Fig. 1).

However, manual fURS requires two surgeons (one manipulating the FU, the assistant operating the EEs), and introduces several challenges for them—namely X-ray exposure, poor ergonomics, a limited workspace, and complex coordination with each other.

Mechanical ureteroscope accessories (UA) such as the *LithoVue Empower*^1^ allow the surgeon to perform some steps in manual fURS procedures (e.g., stone grasping) without assistance [4]. They are compact and—as the surgeon actuates all endoscope DoF—do not affect the haptic feedback from the operation site. The surgeon can still perceive the translational friction with the fingertips manipulating the FU shaft and the resistance against tip bending with the thumb controlling the lever at the FU handle. On the downside, these accessories only support some intervention steps and increase the endoscope weight, which the surgeon must carry during the intervention (Table 1).

**Table 1:** Properties of ureteroscope accessories (UAs), attachable actuation units (AAUs) and telemanipulation systems (TSs) (degrees of property fulfillment: + high, $\circ$ medium, − low; table modified from [5])

Property	UA	AAU	TS
Compactness	+	+	−
Actuation of endoscope DoF	−	$\circ$	+
Weight compensation	−	$\circ$	+
Easy conversion to manual fURS	+	$\circ$	−
Haptic feedback from operation site	+	−	−
Enable solo surgery	$\circ$	$\circ$	−

Several robotic systems for the control of flexible endoscopes have been developed in the past. They can be classified into attachable actuation units (AAUs), which actuate some or all DoFs at the endoscope handle and are either hand-held or mounted on a passive arm, and robotic telemanipulation systems (TSs), which allow the teleoperation of the endoscope from a remote surgeon console (compare [5] for a detailed overview).

AAUs as described in [6–11] are compact, but most do not actuate all endoscope DoFs. Hand-held systems increase the weight carried by the surgeon, while the attachment to a passive arm complicates interventions with frequent adaptations of the endoscope handle pose. During each pose adaptation the combined weight of endoscope, actuation unit and passive arm must be handled, while the passive arm restricts the motion capabilities.

TSs as presented in [12–17] provide control of the endoscope’s (and the EE’s) DoFs and full weight compensation. However, the combined footprint of robot cart and surgeon console is large and they require a second surgeon at the operating room (OR) table. An intraoperative conversion to manual fURS is complex, since it requires sterile clothing of the surgeon, removing the robot cart from the patient and undocking the FU from the robot. The haptic feedback is diminished or lost in both AAUs and TS.

To overcome these shortcomings, we propose the robotic system *CoFlex*, which enables a single surgeon to perform a fURS intervention (solo surgery or SSU). In a previous publication we introduced a first prototype and tested its feasibility for stone grasping with non-medical participants [5].

The work at hand makes the following contributions:

- System enhancements (hardware and software) to allow SSU throughout the complete fURS intervention
- System evaluation in an exploratory user study with non-medical participants and surgeons
- Assessment of the general feasibility of the approach, identification of system optimization potentials and future research directions

## Materials and methods

The following materials and methods were applied in system design and evaluation.

### Materials

The robotic component of *CoFlex* is the versatile robot DLR MIRO, which carries at its tool interface the *robot side unit* (RSU) with the attached FU handle. The *patient side unit* (PSU) is mounted close to the patient. The system workflow is implemented as a state machine, which parametrizes and activates the control states of the robot.

**Fig. 2:**
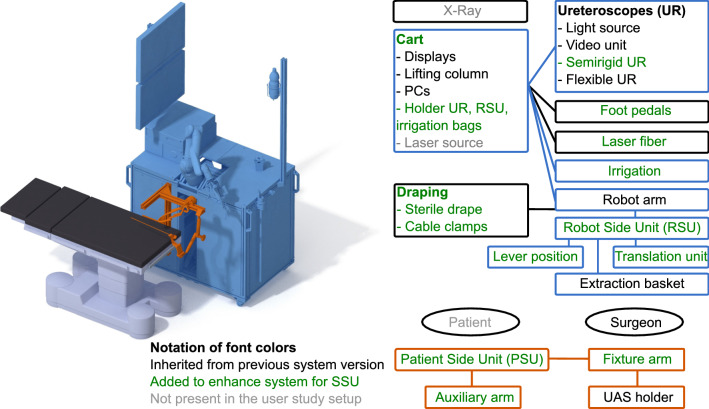
Hardware components (rectangles) and persons (ellipses) involved in a fURS solo surgery procedure. Lines between components represent mechanical connections. The mobile cart (blue) is intraoperatively positioned besides the operating table, the patient side unit (orange) is attached to the side rails of the operating table

A semi-rigid ureteroscope *Olympus WA2UR14A* with camera head *Olympus OTV-S7ProH-FD* and a FU *Olympus URF-V*^2^ were used in system development and evaluation. A dial indicator holder *Hoffmann 440 610 400*^3^ and a gooseneck *Gravity MA Goose L*^4^ serve as fixture and auxiliary arm in the PSU. Two membrane buttons *FTWS-01*^5^ and a position encoder *AMT103-V*^6^ were integrated in the RSU. The disposables for the user study included two *Flexor Ureteral Access Sheaths FUS-120035*, one *Flexor Ureteral Access Sheat FUS-120045* and a stone extractor *NGage NGE-017115*.^7^ Two simulators were applied in the user study: the reachability of kidney calyces was evaluated on silicone models of a left and a right kidney,^8^ the fURS intervention on the *Endo Urology Trainer*.^9^

### Methods

As a surgical robot must also meet non-technical requirements such as clinical need and effectiveness, compatibility with the clinical workflow and usability [18], the development of *CoFlex* pursued an intervention-driven approach. After kidney stone removal by fURS had been identified as a clinically relevant application, *CoFlex* was designed to perform this complete intervention in a laboratory environment. The scope of the exploratory user study was the resource efficient evaluation of the technical and clinical feasibility of the investigated concept.

#### Hardware design

**Fig. 3:**
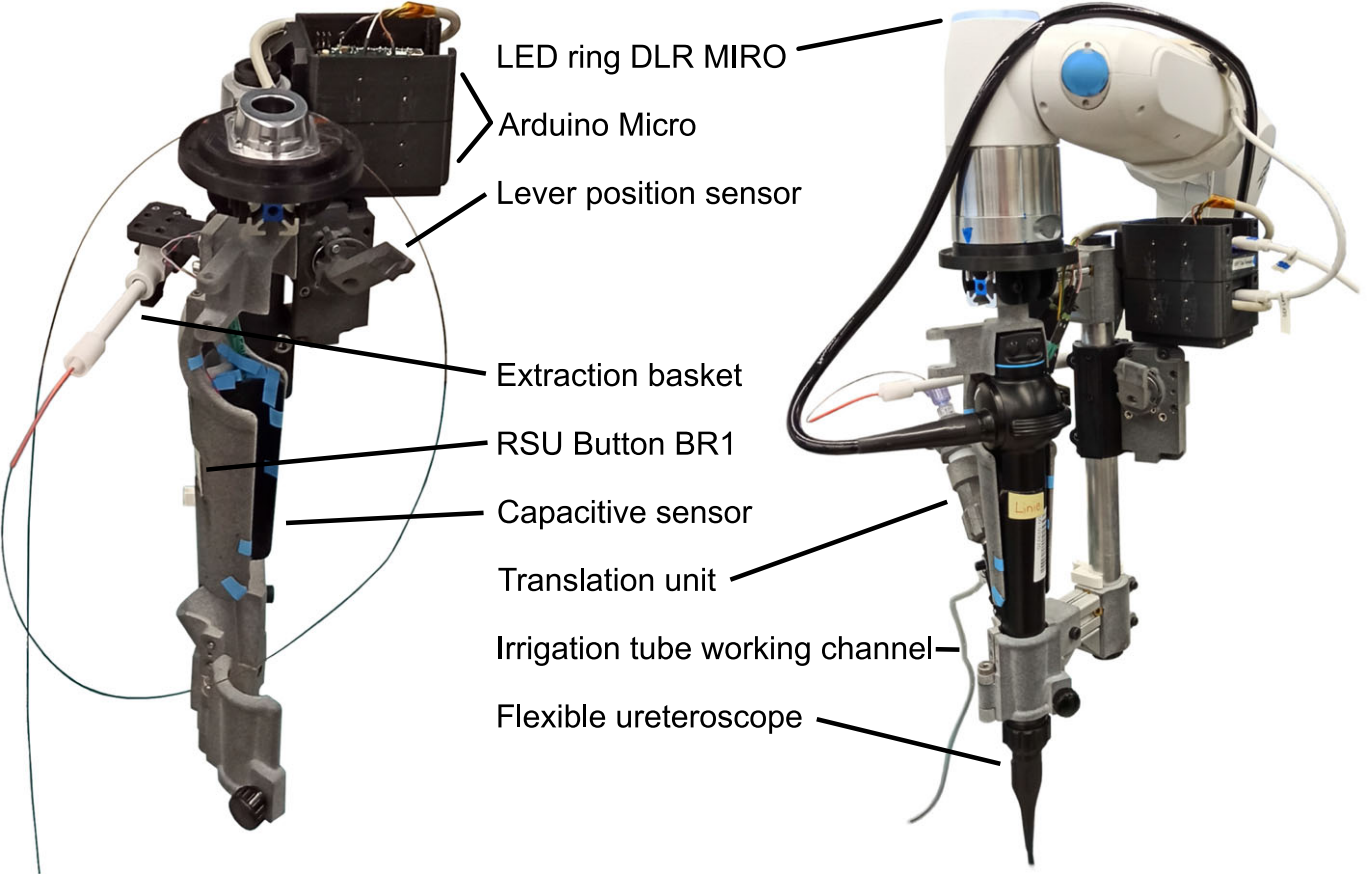
Detail view of the RSU without (left) and with attached FU (right): the extraction basket is clamped to the RSU structure and inserted into the FU working channel via the translation unit. The LED ring informs the surgeon about the system state

**Fig. 4:**
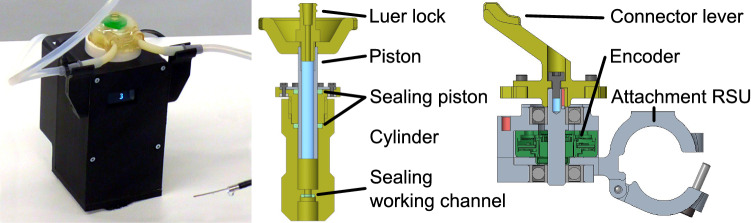
Irrigation pump (left), cross section of the translation unit (middle) and cross section of the position encoder for the lever at the FU handle (right)

The system hardware was enhanced to perform SSU in fURS (see Fig. 2):

- *Mobile cart*: It stores the system components and allows to adapt the robot base in height and tilting.
- *Irrigation*: To enable SSU in fURS, the surgeon has to interact with X-ray machine, laser source and active irrigation. The activation of X-ray and laser by the surgeon via foot pedal is established in clinical practice. An additional foot pedal for active irrigation is not feasible, as active irrigation and laser are often used simultaneously. Thus, we integrated an irrigation pump in the mobile cart (see Fig. 4) and two control buttons in the RSU. Pressing both buttons simultaneously (de)activates the pump, double clicking on one button switches between 5 predefined pump speed levels with flowrates from 0.90 to 1.50 ml/s.
- *PSU*: The PSU structure was extended for fixture to the side rail of the OR table. Since it reaches over the hip of the patient (see Fig. 1), the whole PSU is within the sterile field (which simplifies the system setup) and can be positioned opposite the C-arm to prevent interference with the intraoperative X-Ray imaging. The fixture arm facilitates positioning and fixturing the UAS holder in 5 DoFs. The auxiliary arm, implemented as a gripper at a gooseneck fixture, can grasp the semi-rigid ureteroscope or accessories like guidewires.
- *RSU*: The redesigned RSU (see Fig. 3) provides the tool-free attachment of the FU handle, two irrigation buttons, the lever position sensor (see Fig. 4) and a capacitive sensor to detect the grasping of the RSU by the surgeon.
- *Cable and laser fiber guidance*: Two disposable cable clamps (at the upper and lower arm of the MIRO) ensure defined guidance of the cables to RSU and FU and a quick, tool-free removal of the FU for conversion to manual fURS. The translation unit (see Fig. 4) enables the surgeon to shift the laser fiber or other EEs up to 20 mm using only one hand.

**Table 2:** Data of study participants

ID	Gender	Age	Handedness	Experience robots	Experience FUs	Performed tasks
1	m	57	Both	3	10	2
2	m	39	Right	5	10	2
3	m	40	Right	2	10	2, 3
4	m	37	Right	3	9	2, 3
5	m	33	Right	5	3	2, 3
6	f	44	Right	2	1	1
7	f	27	Right	4	2	1
8	f	25	Right	8	1	1
9	m	60	Right	4	1	1
10	m	27	Left	9	1	1
11	m	26	Both	10	3	1

#### Software architecture

**Fig. 5:**
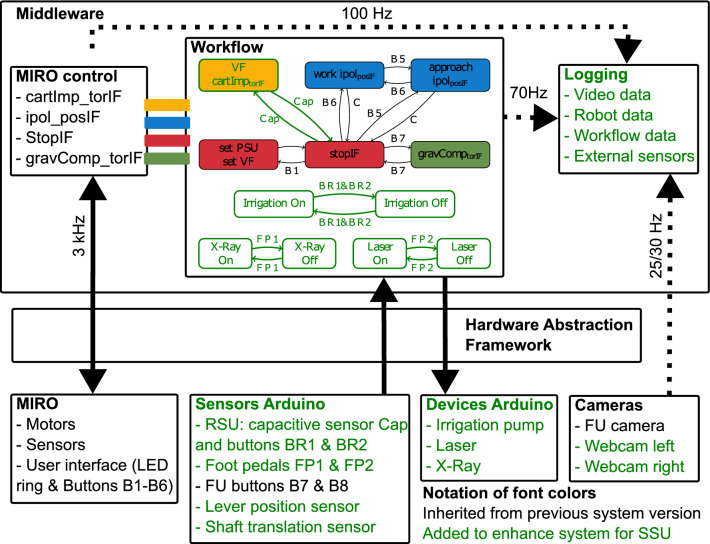
The modular software architecture of *CoFlex*: the workflow is parametrized externally and contains four state machines. One triggers the activation of the different MIRO control modes, the others control the state switching of irrigation, X-ray and laser. The middleware interconnects all system components and facilitates the replacement of components, while the logging allows time-synchronized data acquisition from multiple sources

Figure 5 shows the software architecture of *CoFlex*. The workflow activates the Cartesian impedance controller $cartImp_{torIF}$ for the FU operation triggered by the capacitive sensor in the RSU (instead of a button in [5]). Thus, the surgeon starts and stops the robot motion by grasping and releasing the RSU. This grasp detection allowed a reduction of the minimal friction torques in $cartImp_{torIF}$ to improve the backdrivability of the MIRO. The workflow also interfaces external sensors and devices via microcontrollers *Arduino Micro*.^10^ A time-synchronized logging of data from different sources was implemented. Endoscope camera and webcam data are logged with the video framerates (endoscope 25 Hz, webcams 30 Hz), workflow data are logged with 70 Hz, all other data with 100 Hz.

**Fig. 6:**
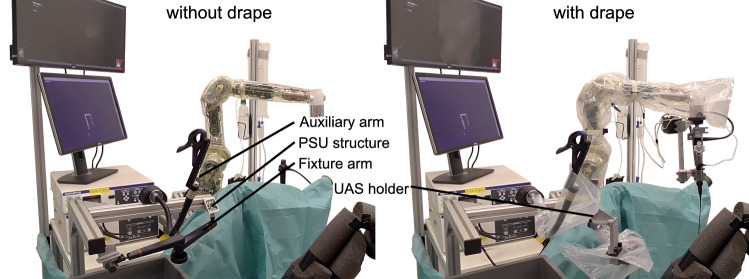
Test setup for Task 1: undraped system with the PSU attached to the OR table side rail (left) and draped system after the setup procedure (right)

### User study design and procedure

The three user study tasks addressed three aspects of robotic fURS. For each trial in each task, the execution time was measured and the participants filled out the NASA-TLX [19] and the System Usability Scale (SUS) [20, 21] questionnaire.^11^

- *Task 1: Setup time added by the robotic system*: Task 1 was evaluated by six non-medical participants (ID 6–11 in Table 2, average age 34.8 ± 14.3 years). On a scale of 1–10, with 1 = none to 10 = expert, they rated their experience in using robots (${\hbox {Exp}}_{\textrm{Rob}}$) as 6.2 ± 3.3, and their experience in using $({\hbox {FUs~Exp}}{\textrm{FU}}$) as 1.5 ± 0.8.
- *Task 2: Reachability of kidney calyces*: The five participating surgeons (ID 1–5, average age 41.2 ± 9.2 years) rated their ${\hbox {Exp}}_{\textrm{Rob}}$ as 3.6 ± 1.3 and their $\hbox {Exp}_{\textrm{FU}}$ as 8.4 ± 3.0.
- *Task 3: Feasibility of SSU in fURS*: Three surgeons (ID 3–5, average age 36.7 ± 3.5 years) executed this task. They rated their ${\hbox {Exp}}_{\textrm{Rob}}$ as 3.3 ± 1.5 and their $\hbox {Exp}_{\textrm{FU}}$ as 7.3 ± 3.8.

#### Procedure task 1

The participants performed the following additional setup steps introduced by the robotic system (two subsequent trials after one demonstration and one training run):

- *PSU attachment to the OR table side rail*: The PSU was carried from the instrument cart to the OR table side rail and mounted there (compare Fig. 6, left).
- *PSU draping*: The fixture arm and the auxiliary arm at the PSU were draped. Subsequently, the UAS holder was attached to the fixture arm.
- *Preparation of the mobile cart*: The MIRO robot was draped, and then, the RSU was attached and connected to the drape. The cable fixtures were positioned on the draped robot. After fixating the FU and connecting the RSU cables to the two microcontroller boards, the FU and RSU cables were clamped in the cable fixtures.
- *Positioning of the mobile cart*: The cart was moved from the marked preparation position to the marked working position at the OR table and two foot pedals were placed in front of the OR table. The system state at the end of Task 1 is shown in Fig. 6, right.

**Fig. 7:**
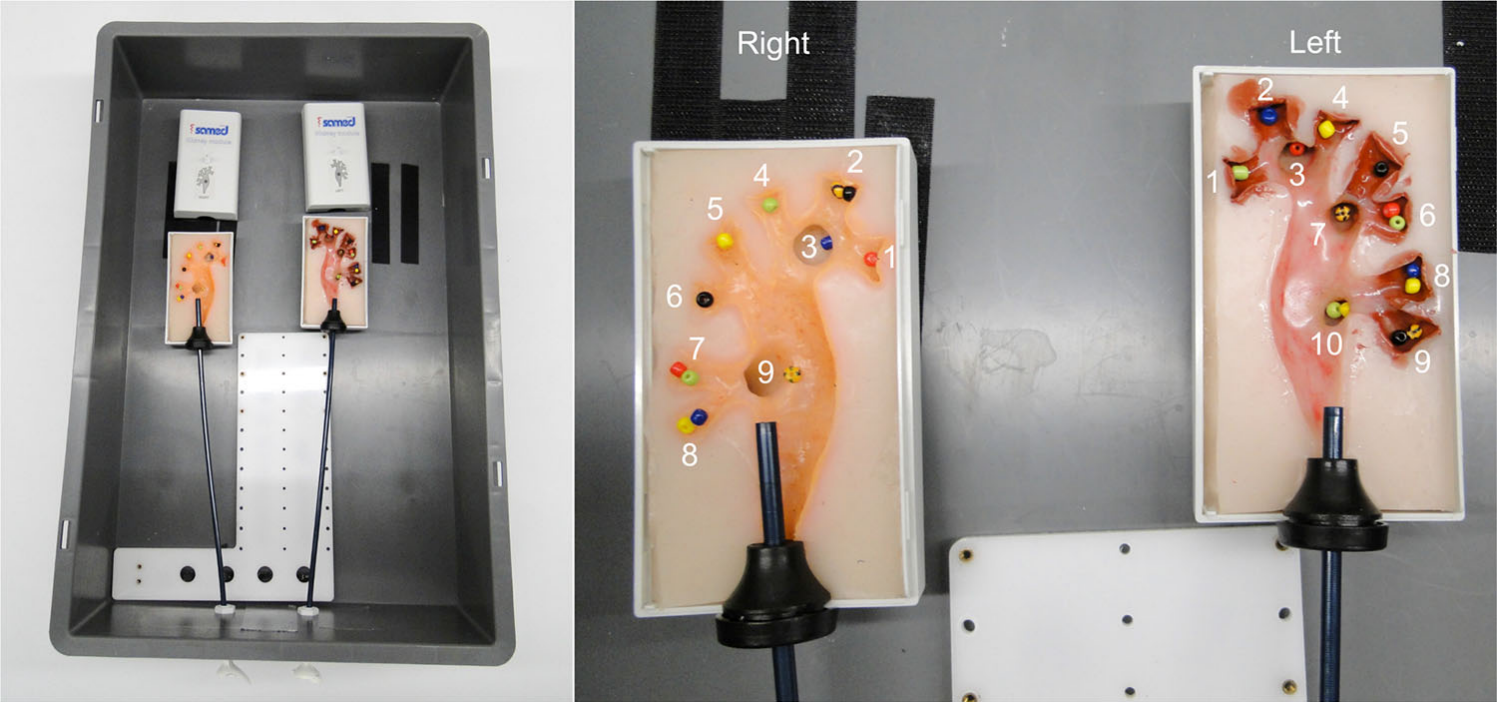
Test setup for Task 2: both kidney phantoms with inserted UAS were positioned in a water-filled plastic box. The calyces were marked by colored pearls

#### Procedure task 2

To investigate the reachability of kidney calyces with *CoFlex*, we reproduced the test setup by Schlager et al.[22] as shown in Fig. 7. Each of the five participants had to inspect 19 calyces in the two SAMED kidney phantoms (9 in the right and 10 in the left kidney, in total 95), which were identifiable by colored pearls within them.

All study participants performed at first the manual scenario (${\hbox {Sc}}_{\textrm{Man}}$) and then the robotic scenario (${\hbox {Sc}}_{\textrm{Rob}}$). Before each measured trial, they could familiarize themselves with the respective scenario for a maximum time of 10  min. Calyces not inspected within the maximum trial time of 600 s counted as missed.

#### Procedure task 3

**Fig. 8:**
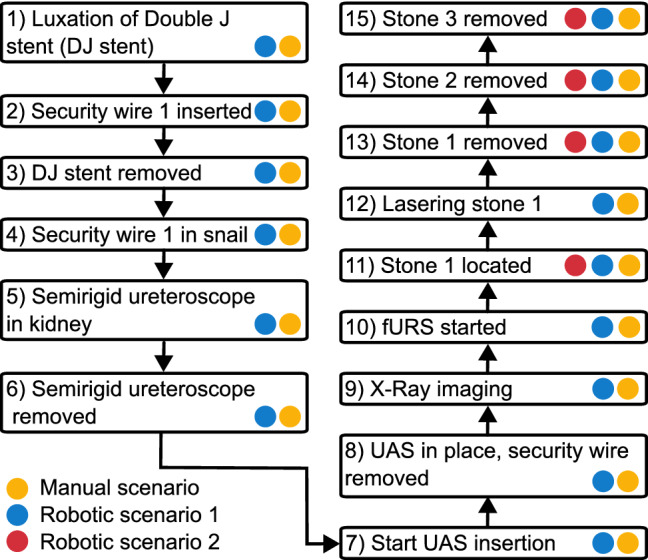
Characteristic steps of Task 3 in the three performed scenarios

A complete fURS intervention was executed to remove three stones (one per calyx group, compare the arrows in Fig. 1, right) from the right kidney of the *Endo Urology Trainer*. The times of the steps in Fig. 8 were recorded. Two manual procedures (steps 1–15; performed by different surgeons but with the same assistant surgeon; (${\hbox {Sc}}_{\textrm{Man}}$)) served as basis for comparison. In the robotic scenario 1 (${\hbox {Sc}}_{\textrm{Rob1}}$), three surgeons conducted complete fURS interventions with the *CoFlex* system. In the robotic scenario 2 (${\hbox {Sc}}_{\textrm{Rob2}}$), they performed only the stone removal with the FU (steps 10, 11, 13, 14, 15) using *CoFlex*.

## User study results

To assess the obtained global NASA-TLX values, we compare them in the following with the percentiles for global TLX-values calculated by Grier: 25th percentile 36.77, 50th percentile 49.93, 75th percentile 60.00 and 90th percentile 68.00 [23].

Table 3 lists the results for Task 1. The improvement in time between trial 1 and 2 probably results from learning effects. With further learning and minor design improvements, an average added setup time below 300 s seems attainable (minimum setup time in the study: 249 s). This is less than 10 % of the 70.2 min total operation time for fURS reported, e.g., by Hein et al.[24].

**Table 3:** Results of Task 1

	Trial 1	Trial 2
Added setup time [s]	363.5 ± 57.7	341.7 ± 71.6
NASA-TLX	31.2 ± 14.9	25.2 ±13.3
SUS	79.2 ± 14.3	82.9 ± 14.4

In both trials, the NASA-TLX values were below the 25th percentile. Attributing adjective ratings to SUS values as suggested by Bangor et al. [21], the usability of the setup procedure (Task 1) is rated good to excellent.

Table 4 summarizes the results for Task 2 with five surgeons. Two of five surgeons in ${\hbox {Sc}}_{\textrm{Rob}}$ and three of five surgeons in ${\hbox {Sc}}_{\textrm{Man}}$ inspected all 19 calyces (see Fig. 7).

**Table 4:** Results of Task 2

	${\hbox {Sc}}_{\textrm{Man}}$	${\hbox {Sc}}_{\textrm{Rob}}$
Time calyx inspection [s]	228.4 ± 133.7	362.6 ± 283.2
NASA-TLX	48.9 ± 20.1	58.1 ± 16.0
SUS	63.6 ± 15.3	51.5 ± 19.9

The ratios of the successfully visualized calyces were 90/95 or 94.74 % for $\hbox {Sc}_{\textrm{Man}}$ and 89/95 or 93.68 % for ${\hbox {Sc}}_{\textrm{Rob}}$. Calyx R8 was missed three times, all other missed calyces only once (${\hbox {Sc}}_{\textrm{Man}}$: R1, R8, R9, L1, L6; ${\hbox {Sc}}_{\textrm{Rob}}$: R3, R6, R8 (twice), L8, L9). This indicates that calyx inspection is possible with *CoFlex*. However, the inspection of calyces requiring maximum FU tip bending like R8 and L9 proved to be challenging in both scenarios. In ${\hbox {Sc}}_{\textrm{Man}}$, the success rate was lower and the average time higher, than the values reported by Schlager et al. for the reusable FU [22]. This might have resulted from the inverted mapping (see subsection “Limitations of the user study”) of the FU in our study. The measured time for the robotic system was comparable to those for commercial single-use FUs like the *Pusen Uscope UE3022* in [22].

**Table 5:** Results of Task 3: complete fURS intervention for manual scenario (n=2 surgeon teams) and robotic scenario 1 (n=3 surgeons), only fURS stone removal for robotic scenario 2 (n=3 surgeons) (compare Fig. 8)(^a^values for the two participants, who removed all three stones)

	${\hbox {Sc}}_{\textrm{Man}}$	${\hbox {{Sc}}}_{\textrm{Rob1}}$	${\hbox {Sc}}_{\textrm{Rob2}}$
Time complete [s]	1173.5 ± 355.7	2131.0 ± $$338.0^{a} $$	X ± X
Time fURS stone removal [s]	506.0 ± 391.7	1126.5 ± $27.6^{a}$	576.7 ± 164.6
NASA-TLX	46.0 ± 7.5	65.2 ± 24.2	47.9 ± 11.3
SUS	65.0 ± 7.1	46.7 ± 12.3	54.2 ± 7.6

The NASA-TLX value approached the 50th percentile for the manual and the 75th percentile for the robotic scenario. The comparatively high workload in ${\hbox {Sc}}_{\textrm{Man}}$ probably results from the inverted mapping. The surgeons rated the usability in ${\hbox {Sc}}_{\textrm{Man}}$ as good and in ${\hbox {Sc}}_{\textrm{Rob}}$ as OK. According to the surgeons’ verbal feedback, the increased task completion time and workload as well as the lower SUS values in ${\hbox {Sc}}_{\textrm{Rob}}$ mainly resulted from workspace limits and friction/mass inertia of the MIRO.

Table 5 presents the results for the manual and the two robotic scenarios of Task 3. In the manual scenario, both surgical teams removed all three stones. In ${\hbox {Sc}}_{\textrm{Rob1}}$ (complete SSU intervention), two of three surgeons removed all three stones. The third surgeon could remove two stones (total time till removal of stone 2 1194 s, time fURS stone removal 730 s) but failed to grasp the 3rd stone with the robotic system. In the subsequent ${\hbox {Sc}}_{\textrm{Rob2}}$ (only stone removal with the FU) all surgeons successfully removed all three stones.

This demonstrates that SSU is possible with the current system. However, the surgeons’ feedback and the missed stone indicate that the workspace for positioning the FU handle should be enlarged. The observed times for both the complete procedure and the fURS stone removal alone were in ${\hbox {Sc}}_{\textrm{Rob1}}$ about twice as high as in ${\hbox {Sc}}_{\textrm{Man}}$. While the time increase for the fURS stone removal mainly resulted from the robot handling and the increased workload for the surgeon, the time increase before fURS might also be caused by limitations of the simulator (see subsection “Limitations of the user study”). In ${\hbox {Sc}}_{\textrm{Rob2}}$, the average time for fURS stone removal was about 50 % of the time in ${\hbox {Sc}}_{\textrm{Rob1}}$ and approximated the time in the manual scenario.

The average NASA-TLX value approached the 90th percentile from [23] in ${\hbox {Sc}}_{\textrm{Rob1}}$, underlining the scenario’s complexity for first-time users. In ${\hbox {Sc}}_{\textrm{Rob2}}$, however, it decreased below the 50th percentile, to a similar level as in the manual scenario. Similar to Task 2, the surgeons rated the usability in the manual scenario as good and in the robotic scenarios as OK. The progression of the times for stone removal, NASA-TLX and SUS values between the robotic scenarios 1 and 2 indicate learning effects already within two trials.

## Discussion

This section discusses the implications of the user study regarding the feasibility of the approach, its limitations, and potentials for system optimization.

### Feasibility of the approach

*CoFlex* provides—like the UA [4] described in the “introduction” section —unaltered haptic feedback from the operation site. In particular the haptic feedback for the FU shaft translation is clinically relevant: when the FU is inserted into the ureter without UAS, an increased translational resistance means a higher load of the ureter wall. This may cause lesions of the ureter wall or even an ureter rupture.

*CoFlex* is technically more complex than UA, but reduces workflow interruptions (e.g., the FU pose is maintained during EE exchange), allows gravity compensation and safety functions like virtual walls. The forces/torques from the virtual walls do not interfere with the haptic feedback as they are injected to the FU handle and felt by the surgeon with the palm of the hand.

**Fig. 9:**
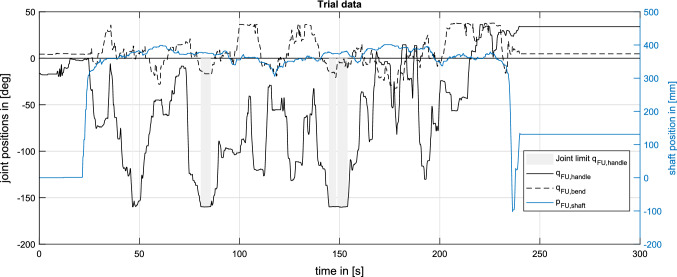
FU motion described by shaft translation $\textrm{p}_{\textrm{FU,shaft}}$, tip bending $\textrm{q}_{\textrm{FU,bend}}$ and rotation around the FU handle axis $\textrm{q}_{\textrm{FU,handle}}$ in a robotic trial of Task 2. The data acquisition from multiple sources (shaft translation sensor, lever position sensor and MIRO axis 7) with the implemented logging infrastructure (compare Fig. 5) allows a detailed understanding of the surgeon–system interaction

Considering the non-technical requirements from from the subsection “Methods”, the user study confirmed *CoFlex’s* feasibility to address the clinical need for SSU in fURS. Additionally, it provided insights regarding the effectiveness, workflow compatibility, and system usability.

The measured times in Tasks 1 and 3 were promising for a prototype. The total surgeon time in ${\hbox {Sc}}_{\textrm{Rob1}}$ was less than the combined time of both surgeons in the manual scenario:

1 $$\begin{aligned} T_{\textrm{CompSc}^{\textrm{Rob1}}}=2131.0~s<2347.0~s=2*T_{\textrm{CompSc}^{\textrm{Man}}} \end{aligned}$$

When inserting the fURS stone removal times from $\hbox {Sc}_{\textrm{Rob2}}$ to account for training effects and the setup time from trial 2 of Task 1, the difference becomes even more distinct:

2 $$\begin{aligned} T_{\textrm{BestCaseRob}}&=T_{\textrm{Setup2nd}}+T_{\textrm{CompSc}_{\textrm{Rob1}}}-T_{\textrm{fURSSc}_{\textrm{Rob1}}}\nonumber \\&\qquad +T_{\textrm{fURSSc}_{\textrm{Rob2}}}\nonumber \\&\quad =(341.7+2131.0-1126.5+576.7)~s\nonumber \\&\quad =1922.9~s<2*T_{\mathrm{CompSc_{ Man}}} \end{aligned}$$

This suggests that derivatives of the developed systems might be able to save a significant amount of surgeon time and thus cost in fURS.

### Limitations of the user study

The number of study participants in the exploratory user study allowed qualitative but no statistically significant results. The inverted mapping (compared to what the surgeons were used to) between lever motion at the handle and tip bending of the applied FU alluded in the section “User study results” probably increased times and workload and decreased the SUS values in both manual and robotic scenarios. The study was performed in a laboratory environment on commercially available ureteroscopy simulators. While the spatial situation in the OR could be reproduced sufficiently, the X-ray machine and laser source were missing in the test setup. This seems acceptable, as the interaction interfaces for the surgeon (laser fiber, two foot pedals for X-Ray and laser activation) were present. The *Endo Urology Trainer* used in Task 3 exhibited an unnatural friction behavior (excessive friction during UAS insertion, unrealistic low friction between Double J/guidewires and ureter). This caused the latter devices to slide out of the simulator, which increased the operation time. In addition, air bubbles in some kidney calyces caused mirror effects.

### Optimization of components

All system components proved functional in the user study. However, the following minor potentials for optimization were identified:

- *Cart*: The display(s) should be positioned over the patient.
- *Irrigation*: Three out of five surgeons suggested to investigate alternatives to the currently implemented button pattern for the control of the active irrigation (see subsection “Hardware design”). An improved sealing concept should be implemented at the translation unit to prevent leakage.
- *PSU*: The weight and storage size of the PSU structure should be reduced. As the knurled screw to stiffen the fixture arm is cumbersome to turn through the sterile drape, alternatives to induce the stiffening should be investigated.
- *RSU*: The LED ring at the MIRO tool interface should be reduced in brightness or covered by opaque material to avoid irritation of the user. As some users had difficulties to reach the slider at the extraction basket handle, it should be repositioned.

## Conclusion and outlook

The robotic system *CoFlex* allows solo surgery in a complete fURS intervention and simultaneously collects quantitative data to support further system development (see Fig. 9). An exploratory user study with non-medical participants and surgeons confirmed the feasibility of the developed system for fURS and its compatibility with the surgical workflow. The observed operation times for a single surgeon were similar to the combined time for both surgeons in manual fURS. Training effects and usability improvements could further reduce operation time. In the future, we aim to implement the measures from "4.3" section and increase the robot’s backdrivability by inertia and friction shaping. We will also further examine the acquired data to improve our understanding of the surgeon–system interaction and optimize the fURS workflow.

## Supplementary Information

Below is the link to the electronic supplementary material.

Supplementary file 1 (mp4 54040 KB)

## Footnotes

1 Boston Scientific, Marlborough, MA, USA.

2 all Olympus Deutschland GmbH, Hamburg, Germany.

3 Hoffmann SE, Munich, Germany.

4 Adam Hall GmbH, Neu-Anspach, Germany.

5 Richard Wöhr GmbH, Höfen, Germany.

6 CUI Devices, Lake Oswego, OR, USA.

7 all CookMedical Inc., Bloomington, IN, USA.

8 SAMED GmbH, Dresden, Germany.

9 MedTecton GmbH, Lucerne, Switzerland.

10 Arduino Srl, Monza, Italy.

11 All participants had signed a declaration of consent before. The obtained data were made anonymous immediately after the end of the user study.
